# Modeling metastasis – leveraging novel tools to streamline discovery in advanced cancer

**DOI:** 10.1242/dmm.052449

**Published:** 2025-09-03

**Authors:** Nicole M. Eskow, Eva Hernando

**Affiliations:** Department of Pathology, NYU School of Medicine, New York, NY 10016, USA

## Abstract

Metastasis remains a leading cause of morbidity and mortality in patients diagnosed with cancer. A variety of *in vitro* and *in vivo* approaches have been employed to study the individual steps of the metastatic cascade. However, these methodologies are sometimes limited in their ability to recapitulate the biological complexity and heterogeneity of human tumor biology. As a result, significant knowledge gaps still exist regarding the development, growth and evolution of treatment resistance in metastatic tumors. In this Perspective, we discuss the benefits and drawbacks of current, widely used techniques to model metastatic disease. We also highlight novel approaches utilized in recent studies to confront the limitations posed by classic modeling techniques. Ultimately, we provide suggestions for ensuring scientific rigor and reproducibility in metastasis studies, and we propose key areas of focus for developing next-generation models of metastasis.

## Teaser

**Summary:** We comprehensively review current cancer metastasis models, highlight advances and gaps, and advocate open data sharing to enhance model selection, experimental rigor and clinical relevance.

## Introduction

When diagnosed at an early stage, cancers are often curable using localized therapies, such as surgery and radiation. However, outcomes are relatively poor for patients with cancers that have metastasized to distant tissues (Welch and Hurst, 2019). While systemic therapies, such as chemotherapy, targeted therapy and immunotherapy, can treat and prolong the lifespan of patients with metastatic cancer, many late-stage cancers are incurable with current treatments and technologies (Gerstberger et al., 2023). Studies to decipher the molecular mechanisms underlying metastasis are critical to our ability to prevent, treat and cure metastatic disease.

Studies to decipher the molecular mechanisms underlying metastasis are critical to our ability to prevent, treat and cure metastatic disease

**Fig. 1.**
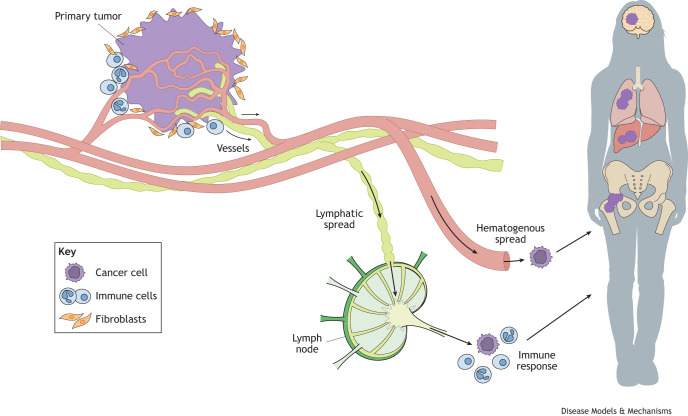
**Metastasis is a complex multi-tissue event.** For tumor cells to metastasize, they must undergo a highly inefficient and demanding process during which they enter the lymphatic and/or hematogenous vasculature and seed a foreign microenvironment. Tumor cells must, therefore, acquire genetic and epigenetic alterations that enable their ability to survive each step of the metastatic cascade, including immune evasion, shear stress and organ-specific adaptations. Original figure produced by Uta Mackensen.

To conduct innovative and impactful research on metastasis, scientists must select model systems that are best aligned with their specific research questions and areas of focus. Metastasis is a highly complex, multi-step process in which cancer cells must invade surrounding tissue, intravasate into the circulatory system, survive, extravasate and proliferate at a distant site (Lambert et al., 2017) – all while enduring substantial physical stress, evading immune detection, and adapting to a foreign microenvironment (Massague and Ganesh, 2021) (Fig. 1). *In vitro* models are powerful tools for dissecting discrete steps of this sequence (Bouchalova and Bouchal, 2022; Malandrino et al., 2018), whereas *in vivo* models can offer a comprehensive, physiological view of metastatic progression (Gomez-Cuadrado et al., 2017). Here, we describe the advantages and limitations posed by commonly used *in vitro* and *in vivo* models of metastasis, together with suggested solutions and alternative approaches, to ultimately advance metastasis research.

## *In vitro* modeling: metastasis in a dish

### Two-dimensional models

*In vitro* systems enable modeling of metastasis in a highly controlled environment. While these systems are unable to recapitulate the multiorgan processes of metastatic disease, they allow scientists to examine discrete steps of the metastatic cascade at a granular level that is typically unachievable *in vivo*. Classically, laboratories have utilized two-dimensional (2D) cultures of tumor cells to measure their intrinsic metastatic capabilities. For example, transwell migration assays have been used to analyze the directional migration and invasion of cancer cells in response to chemoattractants (Bouchalova and Bouchal, 2022); wound-healing and spreading assays have been used to track the physical movement and adhesion dynamics of cells (Pijuan et al., 2019); and matrix degradation assays have been used to characterize mesenchymal versus ameboid migration (Hanniford et al., 2015). Cell lines are typically utilized in these studies; however, continued passaging can result in a significant genetic shift and loss of heterogeneity compared to the original human samples from which they are derived (Quevedo et al., 2020). Multiple laboratories have attempted to address these problems by establishing short-term cultures of human tumor biopsies, helping to preserve the initial biology and heterogeneity of transcriptional cell states in the original tumor (Kleffman et al., 2022; Lai et al., 2012; Rambow et al., 2018). Yet, even under short-term culture conditions, 2D culturing methods fail to recapitulate not only the multicellular makeup but also the physical architecture of human tumors (Guerrero-López et al., 2025). To confront the limitations of using 2D cell line monocultures to study metastasis, three-dimensional (3D) multi-cell culture systems are shedding light on cellular dynamics that cannot be studied using 2D tumor cell cultures alone.

### Organoids

Organoids, often termed ‘mini organs’, are organized 3D cultures designed to mimic the structure and function of human tissue (Zhao et al., 2022). In metastasis research, organoids are often cultured from donor tumor biopsies (Ganesh et al., 2020; Lee et al., 2018). Patient-derived organoids (PDOs) cultured directly from bio-banked human specimens preserve most of the cellular heterogeneity, histological and structural features, and molecular profiles of the patient's original tumor tissue (Porter et al., 2020). As a result, PDOs can be used to examine short-term tumor–immune and tumor–stroma interactions, and provide a platform to test multiple drugs simultaneously against a patient's tumor, which can directly inform their clinical management (Lee et al., 2018). Recently, patient-matched PDOs developed for normal colon, primary and metastatic colorectal cancer, revealed that metastatic cells exhibit greater cell-intrinsic plasticity than the corresponding primary tumor cells, facilitating their ability to adopt unique, non-canonical transcriptional programs (Moorman et al., 2024).

Despite the translational value of PDOs, their widespread development and use is limited to research groups with sufficient access to primary human tissue. In addition, longer-term culturing of PDOs may inadvertently cause disproportionate outgrowth of one or several dominant cancer cell clones (Hebert et al., 2023). Additionally, PDOs are often optimized to sustain the growth of cancer cells over immune or stromal cells, limiting long-term reconstitution of the full tumor microenvironment (LeSavage et al., 2022; Thorel et al., 2024). To overcome these limitations, many experts in PDO development have called for greater standardization of organoid methodology across groups (LeSavage et al., 2022). Researchers are also developing strategies, such as autologous immune cell supplementation, to replenish cell populations that may be lost in PDO cultures (Votanopoulos et al., 2020). Other groups are using pluripotent stem-cell-derived organoid systems to circumvent the need for patient samples (da Silva et al., 2018; Gomez et al., 2019). Although current stem-cell-derived systems may not mimic the full extent of microenvironmental and tumoral heterogeneity seen in patients, they enable experimental standardization and manipulation that is difficult to achieve in PDOs (Kim et al., 2020). The limitations of PDOs can also be partially bypassed by using mouse-derived organoids. Unlike human tissue, mouse-derived systems can be used for complex genetic engineering, high-throughput assays and longer term immunological readouts (Hai et al., 2020; Pan et al., 2023; Tong et al., 2024).

### Organotypic cultures

Beyond organoids, many tumor biologists have turned to organotypic cultures, in which tissue specimens from mouse models are placed directly into culture with minimal processing or digestion. Therefore, organotypic culturing can preserve the architecture and cellular makeup of the original tissue (Spennati et al., 2021). In one study, organotypic cultures of mouse brains bearing metastatic tumors demonstrated that cancer cells can mimic and compete with capillary pericytes to co-opt the brain's vasculature (Er et al., 2018). This brain metastasis organotypic slice model has been further developed as a robust *ex vivo* tool for drug discovery (Steindl and Valiente, 2024; Zhu et al., 2023). Analogous protocols for human organotypic cultures, known as patient-derived explants, are also being developed (Powley et al., 2020; Vaira et al., 2010). Organotypic culturing can be technically challenging – tissue harvesting can induce stress responses and compromise viability (Bak et al., 2024). With this in mind, we believe it presents an appealing future alternative to PDOs.

### Intravasation and extravasation modeling

Organoids and organotypic cultures are excellent models for studying tumors biopsied either from their primary site or distant metastatic sites. Consequently, they often do not capture the dynamic and physically demanding process in which cells intravasate from the primary tumor, travel through the circulatory system and extravasate into a distant organ (Gomez-Cuadrado et al., 2017). To understand these initial steps of metastatic tumor formation, scientists have developed novel 3D microfluidics devices that incorporate multiple stromal, endothelial and immune cell types to mirror the physiological morphology, flow rates and wall shear stresses in capillary systems (Campisi et al., 2018; Virumbrales-Munoz et al., 2019). For example, microfluidics devices can be used to model microvascular systems, such as the blood–brain barrier, enabling the study of the individual contributions of brain stromal cells, such as pericytes and astrocytes, to cancer cell extravasation (Hajal et al., 2021). Alternative chemo-mechanical models predict how microenvironmental stiffness can impact cancer cell morphology and energetics (Jaganathan et al., 2024 preprint). These models effectively complement *in vivo* techniques to study intravasation and extravasation events, such as two-photon microscopy for the quantification of extravasation and cell-cell contact events (Dalla et al., 2024). Ultimately, deeper analyses of the early steps of dissemination will advance our understanding of metastasis by facilitating the identification and characterization of molecular drivers of metastasis. Such studies with microfluidics and chemo-mechanical models may yield biomarkers and therapeutic targets to prevent metastasis in patients with cancer.

[…] humanized models can be labor-intensive [but] we believe that they will elevate metastasis research […]

## *In vivo* modeling: understanding metastasis as a multi-step process

### Genetically engineered mouse models

For decades, murine preclinical models of metastasis have provided a critical foundation for the discovery of therapies to combat advanced-stage cancers. Genetically engineered mouse models (GEMMs) are most often developed in immune-competent mice and allow scientists to explore the metastatic cascade from the first step of tumor initiation (Table 1) (Gomez-Cuadrado et al., 2017). While most tumorigenic GEMMs do not generate metastasis, several models can develop metastasis in the liver, lungs and lymph nodes (Rampetsreiter et al., 2011). Currently, the range of organs to which GEMM tumors can metastasize is limited; for example, there are no known GEMMs that reliably develop brain metastases (Valiente et al., 2018). Alternative strategies include retroviral delivery models, which have been used to investigate the role of mutant AKT1 in promoting metastasis in the non-metastatic BRAF^V600E^/Cdkn2a^Null^ melanoma model (Cho et al., 2015) (Table 1). This strategy generates metastasis in various organs, including the brain, to an extent not previously seen in melanoma GEMMs (Cho et al., 2015). We believe GEMMs offer an excellent platform for investigating all sequential steps of metastasis *in vivo* and elucidating the tumor cell-intrinsic and -extrinsic adaptations that enable metastatic progression. Moreover, GEMMs can provide valuable tools for testing novel therapies and interventions targeting distinct stages of metastasis. Future efforts will focus on further engineering these models to study specific metastatic sites of interest, such as the brain and bone.

**Table 1.** Advantages and limitations of various syngeneic modeling approaches

**Model type**	**Advantages**	**Limitations**
Genetically engineered mouse models (GEMMs)	• Recapitulates the full metastatic cascade	• Most do not produce metastases
		• Metastatic models may not generate tumors at sites of clinical interest, such as brain and bone
Virally-induced models	• Recapitulates the full metastatic cascade	• Highly complex experimental scheme
Transplantation models	• Highly controlled experimental scheme	• Misses the initial steps of metastasis
	• Can directly target metastatic sites of interest	• Lacks intratumoral heterogeneity
		• Limited genetic complexity
		• Often poorly immunogenic

### Transplantation mouse models

Historically, most mouse models of metastasis have involved the injection of human tumor cell lines into immunocompromised mice to generate xenografts. However, these extensively passaged and transformed cells do not accurately reflect the heterogeneity and complexity of human tumors. Patient-derived xenografts (PDX) and PDO transplantation models can overcome this issue (Patton et al., 2021a). Interestingly, PDX have been shown to mimic the metastatic potential of their original tumors upon transplantation into severely immunocompromised NOD-*scid* IL2Rgamma^null^ mice (Piskounova et al., 2015). Yet, these immunocompromised models fall short for their inability to recapitulate the complete immune response in human cancers (Giacobbe and Abate-Shen, 2021). Such models do not permit adequate studies of how the immune system and tumor microenvironment may either block or facilitate metastatic dissemination and growth. Thus, many laboratories now use syngeneic models, or allografts, in which mouse cancer cell lines are injected into immune-competent mice with a matched genetic background (Giacobbe and Abate-Shen, 2021).

Unlike immunocompromised models, syngeneic transplantation models allow interrogation of phenotypes associated with adaptive immunity (Table 1). The efficacy of immune checkpoint inhibitors, such as anti-PD1 and anti-LAG3, against advanced cancers has underscored the importance of considering all components of the immune system in metastasis studies (Tawbi et al., 2022; Wolchok et al., 2025). Nevertheless, many scientists question the clinical translatability of syngeneic models, as they are a fully murine system with no inclusion of human-derived cells. Cell lines for syngeneic modeling are typically derived from aggressive spontaneous tumors or GEMMs bearing numerous oncogenic mutations (Aktary et al., 2023; Connolly et al., 2022). Consequently, tumorigenesis in these GEMMs occurs at an artificially rapid pace, giving rise to homogenous tumors – and subsequent derived cell lines – that are substantially more aggressive and genomically stable, as well as less immunogenic, than human cancers (Connolly et al., 2022; Lonberg, 2025). To increase the immunogenicity of GEMM-derived models, technologies, such as novel inversion-induced joined neoantigen (NINJA), can induce neoantigen expression in mouse models in a highly controlled manner, permitting stronger T-cell responses (Damo et al., 2021). Likewise, the Jedi mouse, in which T cells recognize GFP as their cognate antigen, enables studies of immune escape and its contribution to tumor progression (Agudo et al., 2015). These mice can be used in combination with GFP-expressing murine lines to interrogate how antigen-specific T cells interact with disseminated tumor cells. Carcinogen-induced murine cell lines can also partially address immunogenicity issues via increased heterogeneity and antigenicity – one such example is the UVB-irradiated YUMMER1.7 mouse melanoma line, which exhibits high somatic mutational burden (Wang et al., 2017). However, these carcinogen-induced models often lack the genetic alterations (e.g. chromosomal instability) that commonly occur in corresponding human tumors (Sewduth and Georgelou, 2024).

To bridge the gap between syngeneic and immunocompromised (human cell-based) models, many tumor biologists and immunologists have designed humanized mouse models, in which human peripheral blood mononuclear cells or hematopoietic stem cells are implanted into immunocompromised mice (Cogels et al., 2021). Advanced humanized mouse models have been developed in which implanted human immune cells are supported by the targeted and sequential delivery of cytokine factors via transgenes encoded in DNA-based vectors (Somasundaram et al., 2021). Use of this model enabled the authors to discover a population of tumor-infiltrating mast cells capable of promoting anti-PD1 therapy resistance (Somasundaram et al., 2021). While these humanized models can be labor-intensive and require access to patient samples, we believe that they will elevate metastasis research by enabling studies of human tumor cells in an immune-competent system, increasing the translatability of research findings and encouraging more immunological characterization of metastatic (versus primary) tumor models.

**Fig. 2.**
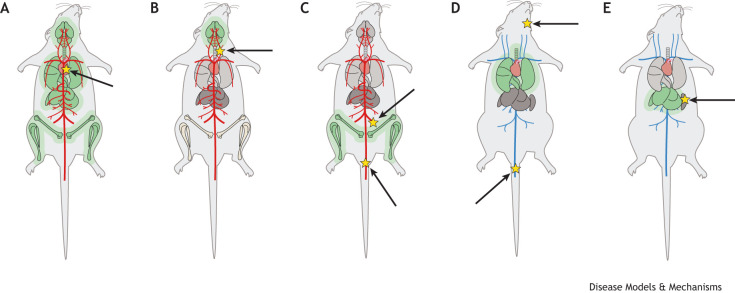
**Tumor cell inoculation routes for transplantation models.** (A-E) Schematics showing injection sites (yellow star) at arterial (red blood vessels; A-C) and venous (blue blood vessels; D,E) locations to yield site-specific metastasis. Intracardiac injection (A), i.e. into the left heart ventricle of tumor cells, yields widespread metastasis across the body, whereas intracarotid (B) and intra-caudal or intra-iliac (C) injections yield metastasis only in the brain and bone, respectively (Balathasan et al., 2013; Kuchimaru et al., 2018; Yu et al., 2016; Zhang et al., 2017). Several routes of venous injection generate lung metastasis, including tail vein and retro-orbital injections (D). Injection of tumor cells into various locations within the portal circulation, such as the splenic vein (E), yield high rates of liver metastasis (Lucke et al., 2024). Injection sites are indicated by arrows and yellow stars; respective metastatic sites are highlighted in green.

Another key variable to consider in transplantation models is the anatomical location into which cells are injected (Fig. 2, Table 2). In some studies, cancer cells are injected directly into the metastatic organ of interest. This approach can enable high-throughput, reproducible studies of the interplay between metastatic cancer cells and the target microenvironment, while also allowing precise control over the anatomical site of tumor initiation (Messmer et al., 2024). However, this technique bypasses almost the entire metastatic cascade. Likewise, the inoculation of thousands of tumor cells into an organ does not recapitulate the process of clonal selection that precedes metastatic tumor formation (Hebert et al., 2023). Some organs may also be sensitive to direct injection methods. For example, intracranial injection of cancer cells can induce severe neuroinflammation, confounding studies meant to dissect tumor–immune and tumor–stroma interactions in the brain (Miarka and Valiente, 2021). Therefore, it is crucial to prioritize methods that do not cause significant and artifactual perturbations of the metastatic sites of interest.

**Table 2.** Advantage and limitations of various routes of tumor instillation to model metastasis

**Route of injection**	**Advantages**	**Limitations**
**Orthotopic injection** into the primary site, sometimes followed by survival surgery or treatment to prolong latency	• Recapitulates nearly the entire metastatic cascade	• May require a long timeline for generation of metastasis
		• Few models reach distal organs after orthotopic implantations (e.g. most will grow too much locally before metastasis)
**Intracardiac injection** (left ventricle)	• High yield of widely disseminated metastasis	• Misses the early steps of primary tumor invasion and intravasation
	• Ultrasound imaging can ensure accuracy of tumor cell delivery	• Extensive optimization and serial injections may be required to increase incidence at specific metastatic sites of interest and prevent metastasis at other sites
		• Tumor cell implantation and growth at undesired sites, such as within the cardiac muscle, may lead to premature death of the animal
**Localized circulatory injection** (carotid artery for brain metastasis, iliac artery for bone metastasis, tail vein for lung metastasis, splenic vein for liver metastasis)	• High yield of metastasis at site(s) of interest	• Misses the early steps of primary tumor invasion and intravasation
		• May require complex surgical procedures, potentially limiting precise tumor cell delivery as well as animal health and viability
**Direct inoculation of the metastatic site** (e.g. cisterna magna for leptomeningeal disease, intratibial, intracranial)	• Highly controlled scheme; ensures equivalent seeding of metastatic organs of interest	• Misses nearly all steps of the metastatic cascade
		• Induces local injury and inflammation
		• Rapid disease progression; narrow window for therapeutic intervention
		• May entail complex, lengthy surgical procedures

To generate metastasis in mice, many scientists inject tumor cells directly into the vasculature. For example, intracardiac injection of cancer cells can yield metastasis across many organs, including the liver, kidneys and brain, whereas tail vein injections are a reliable method for generating lung metastases (Fig. 2; Balathasan et al., 2013; Lucke et al., 2024). While injection into the vasculature is an effective method of metastasis development, it bypasses cancer cell invasion and intravasation from the primary site, and it involves the introduction of an artificially high number of cancer cells into the circulation (Hebert et al., 2023). To address these concerns, cancer cells are often injected into their site of origin, termed orthotopic injection (Table 2). While this modeling skips the initial steps of tumorigenesis, it captures the full process of cells metastasizing from their primary site to distant organs. Consequently, orthotopic models provide an appropriate platform to characterize intravasation events and circulating tumor cells (Gomez-Cuadrado et al., 2017; Kitz et al., 2018). Orthotopic models can also be used to study metastatic steps that involve the lymphatic system rather than the hematogenous vasculature (Reticker-Flynn et al., 2022). In some cases, orthotopic models can be restricted in their metastatic pattern and may not reliably spread to organs of interest, such as the brain (Lee and Yang, 2023). To increase the yield of certain types of metastasis, scientists often perform serial passaging of tumor cells in mice to enhance their tropism for select organs. For example, serial injections were used to develop a variant of the 4T1 syngeneic model with high selectivity towards formation of brain metastases from the mammary gland compared to parental 4T1 cells (Kim et al., 2018). Alternatively, the orthotopic tumor can be surgically resected once it has achieved a certain size, prolonging the survival of the animal and increasing the likelihood of metastasis development (Liu et al., 2024).

### Quantifying metastatic burden

Another outstanding issue in the field is how best to quantify metastatic burden in mice following an experimental intervention. Most commonly, metastasis is quantified by *in vivo* or *ex vivo* imaging of reporter gene products, such as fluorescent proteins and luciferase (Jin et al., 2020). However, these proteins are known to promote a potent immune response in immune-competent hosts, and their expression may vary among individual cells and tumors, possibly reducing metastatic burden and confounding results (Grzelak et al., 2022). We, thus, support the use of alternative high-resolution methods, such as micro-MRI, histological imaging and specialized stains, such as immunohistochemistry, to target tumor-specific markers, or NuMA, which aids detection of human cancer cells in murine hosts (Kleffman et al., 2022; Ma et al., 2023; Monteiro et al., 2022). Yet, such methods can be slow, labor-intensive and prone to sampling bias. Downstream data analysis often requires additional expertise, but digital and AI-based methods can help streamline these analyses. Many software tools, such as Lunit (Brattoli et al., 2024) and PathAI (Dacic et al., 2024), can aid in patient diagnostics, and others, such as HALO (Esce et al., 2024), are designed for broad image analysis applications. Mouse-specific technologies are also becoming available, with one recent publication featuring a tool allowing quantification of melanoma metastasis burden in liver and brain by hematoxylin and eosin staining, with minimal user input (Karz et al., 2025).

### Zebrafish: an alternative to mice

Zebrafish represent a valid and cost-effective non-mammalian *in vivo* model for studying metastasis owing to their ease of genetic manipulation, optical transparency and rapid development (Patton et al., 2021b). Transplantation models in embryonic and adult zebrafish are highly efficient platforms for mechanistic studies and drug screens (Haney et al., 2020; Sturtzel et al., 2023), and genetically engineered zebrafish lines can enable investigations of clinically relevant mutations and tumor heterogeneity (Ju et al., 2009; Langenau et al., 2005). In one study, cell lines generated in BRAF^V600E^; p53^−/−^ zebrafish were transplanted into transparent *casper* zebrafish to investigate differential proliferative and invasive subpopulations in melanoma metastasis (Campbell et al., 2021). The authors showed that, in both zebrafish and patient samples, proliferative cells surrounded invasive cells to form heterotypic clusters, facilitating co-extravasation of both cell populations at metastatic sites (Campbell et al., 2021; White et al., 2008). We believe that further implementation of non-murine *in vivo* models represents an exciting new avenue for high-resolution, high-throughput studies of metastatic mechanisms that cannot be achieved in mouse models alone.

[...] further implementation of non-murine *in vivo* models represents an exciting new avenue for high-resolution, high-throughput studies of metastatic mechanisms [...]

## Looking forward

In this Perspective, we have highlighted the respective advantages and disadvantages associated with current models of metastasis. To ensure rigor and reproducibility when utilizing these models, we have compiled a list of ‘best practices’ to employ in studies of metastasis (Box 1).

Box 1. Best practices for metastasis modeling *In vitro* models

- Prioritize PDOs or short-term (low-passage) culture systems over cell lines.
- Utilize 3D systems (spheroids, organoids) over 2D systems when possible. When use of 3D systems is not possible, employ a variety of 2D culture systems or cell lines to ensure reproducibility across models.
- Choose models with clinically relevant genetic drivers and/or transcriptional cell states.
- Conduct complementary studies in human cells or samples to avoid overreliance on mouse models.

*In vivo* models

- Use at least two to three models per study; avoid overreliance on a single model.
  – Incorporate at least one syngeneic model and one immunocompromised model, when appropriate, to consider immune system contributions.
  – Consider age and sex as experimental variables.
- When possible, prioritize models that recapitulate as much of the metastatic cascade (i.e. orthotopic models, GEMMs), while still being appropriately tailored to the scientific question of interest.
- Use at least two methods for targeting genes of interest.
  – Genetic knockdown: shRNA, sgRNA, transgenic knockout mice.
  – Protein blockade with antibodies or pharmacological compounds.
- Consider reporter gene-independent quantification methods when measuring metastatic burden.

General recommendations

- Prioritize models based on the scientific question of interest.
  – Cell lines are powerful tools for molecular and biochemical discoveries, whereas mouse models permit for studies of metastatic phenotypes at a multi-system level.
- Promote and partake in open data sharing with all selected model systems and encourage collaborations.
- Avoid overreliance on one or two methodological approaches and employ a variety of models and measurements to answer questions.

Beyond the topics discussed here, many questions remain in the metastasis field that require further development and refinement of models. For example, the ability of metastatic tumor cells to establish and, eventually, escape dormancy is a poorly understood process. Some current GEMM and transplantation models exhibit tumor cell dormancy and, therefore, provide a foundation for dormancy-related studies (Nobre et al., 2021). Future models must incorporate a latency phase to allow for closer interrogation of this process (Massague and Obenauf, 2016). Tumors generated from GEMMs and transplantation models progress at a fast pace, with many of these mice achieving a humane endpoint 2–4 months post induction (Dupage and Jacks, 2013). The aggressiveness of these models differs significantly from the prolonged timeline and stepwise progression of tumorigenesis and metastasis in humans. This aggressiveness can also limit the intratumoral heterogeneity observed in these models (Day et al., 2015; Hidalgo et al., 2014). We believe that the next generation of tumor models should be developed to optimize temporal variables in order to model the sequential acquisition of genetic and epigenetic alterations. Such efforts are likely to reveal previously undiscovered facets of tumor development and metastatic progression.

We also encourage scientists to leverage technological advancements in single-cell RNA sequencing [e.g. 10× Genomics, PIP-seq (Clark et al., 2023)], spatial transcriptomics (e.g. 10× Genomics Visium, CosMx, Xenium), proteomics [e.g. PhenoCycler, formerly known as CODEX (Goltsev et al., 2018)], or combined transcriptomics and proteomics [e.g. CITE-seq (Stoeckius et al., 2017)] to characterize their optimized metastasis models. The distribution of such high-resolution data to examine the metastatic niche before, during and after tumor seeding can prove a valuable resource for the field, especially when the models used to generate these datasets are made widely available. Likewise, advances in barcoding and sequencing techniques can and should be utilized to trace metastatic clones in xenograft and GEMM models. These approaches have proven highly valuable in understanding the evolution of primary tumors and can be easily translated to study clonal selection and cellular adaptation in metastasis (Karras et al., 2022; Neftel et al., 2019; Oren et al., 2021).

Overall, we are enthusiastic about the vast opportunities that exist for model development and discovery in the field of metastasis. By improving and distributing current models, we will enhance the translational value of our studies, ultimately serving to improve the management and outcomes of patients with late-stage cancers.
